# Temporal and Sex-Dependent *N*-Glycosylation Dynamics in Rat Serum

**DOI:** 10.3390/ijms26157266

**Published:** 2025-07-27

**Authors:** Hirokazu Yagi, Sachiko Kondo, Reiko Murakami, Rina Yogo, Saeko Yanaka, Fumiko Umezawa, Maho Yagi-Utsumi, Akihiro Fujita, Masako Okina, Yutaka Hashimoto, Yuji Hotta, Yoichi Kato, Kazuki Nakajima, Jun-ichi Furukawa, Koichi Kato

**Affiliations:** 1 Graduate School of Pharmaceutical Sciences, Nagoya City University, 3-1 Tanabe-dori, Mizuho-ku, Nagoya 467-8603, Japan; ksachiko@ims.ac.jp (S.K.); rina03kk@gmail.com (R.Y.); saeko-yanaka@ims.ac.jp (S.Y.); c212803@ed.nagoya-cu.ac.jp (F.U.); mahoyagi@phar.nagoya-cu.ac.jp (M.Y.-U.); yhotta@phar.nagoya-cu.ac.jp (Y.H.); 2 Exploratory Research Center on Life and Living Systems (ExCELLS), National Institutes of Natural Sciences, 5-1 Higashiyama, Myodaiji-cho, Okazaki 444-8787, Japan; 3 Institute for Glyco-Core Research (iGCORE), Gifu University, 1-1 Yanagido, Gifu 501-1193, Japan; taniyama.reiko.w6@f.gifu-u.ac.jp (R.M.); nakajima.kazuki.x0@f.gifu-u.ac.jp (K.N.); 4 Institute for Glyco-Core Research (iGCORE), Nagoya University, Furo-cho, Chikusa-ku, Nagoya 464-8601, Japan; fujita.akihiro.j0@f.mail.nagoya-u.ac.jp (A.F.); furukawa.junichi.n0@f.mail.nagoya-u.ac.jp (J.-i.F.); 5 Graduate School of Medical Sciences, Nagoya City University, 1 Kawasumi, Mizuho-cho, Mizuho-ku, Nagoya 467-8601, Japan; okina@med.nagoya-cu.ac.jp (M.O.); hashimot@med.nagoya-cu.ac.jp (Y.H.); kato.41@med.nagoya-cu.ac.jp (Y.K.); 6 Institute for Molecular Science, National Institutes of Natural Sciences, 5-1 Higashiyama, Myodaiji-cho, Okazaki 444-8787, Japan

**Keywords:** glycomics, glycoproteomics, immunoglobulin G, *O*-acetylation, rat serum, sialylation

## Abstract

We conducted systematic glycomic and glycoproteomic profiling to characterize the dynamic *N*-glycosylation landscape of rat serum, with particular focus on sex- and time-dependent variations. MALDI-TOF-MS analysis revealed that rat serum *N*-glycans are predominantly biantennary, disialylated complex-type structures with extensive *O*-acetylation of Neu5Ac residues, especially in females. LC-MS/MS-based glycoproteomic analysis of albumin/IgG-depleted serum identified 87 glycoproteins enriched in protease inhibitors (e.g., serine protease inhibitor A3K) and immune-related proteins such as complement C3. Temporal analyses revealed stable sialylation in males but pronounced daily fluctuations in females, suggesting hormonal influence. Neu5Gc-containing glycans were rare and mainly derived from residual IgG, as confirmed by glycomic analysis. In contrast to liver-derived glycoproteins, purified IgG exhibited Neu5Gc-only sialylation without *O*-acetylation, underscoring distinct sialylation profiles characteristic of B cell-derived glycoproteins. Region-specific glycosylation patterns were observed in IgG, with the Fab region carrying more disialylated structures than Fc. These findings highlight cell-type and sex-specific differences in sialylation patterns between hepatic and immune tissues, with implications for hormonal regulation and biomarker research. This study provides a valuable dataset on rat serum glycoproteins and underscores the distinctive glycosylation features of rats, reinforcing their utility as model organisms in glycobiology and disease research.

## 1. Introduction

The omics-based approaches that emerged at the end of the last century have promoted the comprehensive identification of the biomolecules that constitute living organisms. In particular, the development of mass spectrometry (MS)-based technologies, such as tandem and multistage MS (MS^n^), has led to the description of glycans covalently attached to proteins (glycome) and further to the comprehensive identification of proteins modified with glycans (glycoproteome) in many species, including humans, mice, plants, fungi, and bacteria [1,2,3]. In particular, glycomic/glycoproteomic studies on model organisms contribute to basic biology by facilitating the understanding of biological phenomena such as development, differentiation, and evolution. Given that glycosylation is dependent on pathological states, glycomic and glycoproteomic studies of human serum and various tissues could be leveraged to discover biomarkers for different diseases and ageing [4,5,6]. This is exemplified by recent network analysis of serum glycoproteome [7,8], which offers insights into the molecular processes underlying the progression of cancer and its potential biomarkers.

Rodents are widely used as models of human pathologies and ageing studies, as well as in drug discovery. Indeed, the mammalian genome was first explored in mice, followed by humans and rats. Compared to mice, rats have larger bodies that are tolerant to surgical procedures and functional assessments. Rat organs are also of substantial size, which enables sampling sufficient amounts of tissues, blood, or other biospecimen. In specific fields, rats are preferable to other experimental animals due to the physiological similarities shared with humans. Furthermore, rats possess larger brains than mice, rendering them particularly well-suited for behavioral studies. Indeed, rats are widely employed as model animals in a range of studies encompassing cancer, diabetes, cardiovascular diseases, neurological disorders, and drug discovery. Their substantial physiological similarity to humans, which outpaces mice in mirroring human pathophysiology, makes rats an advantageous choice as model animals.

Systematic glycomic and glycoproteomic studies have been performed on mouse sera and tissues across various strains and disease models [9,10,11]. In contrast, analogous research focusing on rat serum and tissues remains limited, often restricted to total *N*-glycan profiling from serum glycoproteins or IgG [12,13,14]. Nevertheless, these studies have consistently demonstrated a distinctive feature of rat serum *N*-glycans—namely, the unusually high abundance (40–60%) of *O*-acetylated derivatives of *N*-acetylneuraminic acid (Neu5Ac), which are scarcely observed in mice and virtually absent in humans. Liu et al. recently reinforced this finding through systematic cross-species comparisons, showing markedly elevated *O*-acetylation levels in rats relative to mice and a complete absence in humans [15].

In addition to pathological states, glycan profiles are also influenced by physiological conditions, including sex differences, pregnancy, childbirth, and ageing. Considering the aforementioned physiological and biochemical homology between rats and humans, it is crucial to establish how robust the rat glycome is under such physiological variations, as this would lay the groundwork for future studies that utilize rat models of human pathophysiologies. However, such insights are still scarce.

In this study, we aimed to clarify how sex and daily physiological rhythms influence serum *N*-glycosylation in rats. First, we profiled the sex-dependent and temporal dynamics of the overall serum *N*-glycome. Second, we sought to identify specific glycoproteins responsible for these variations using LC-MS/MS-based glycoproteomic analysis of albumin/IgG-depleted serum. Third, to aid the interpretation of sialylation dynamics observed in non-IgG serum glycoproteins, we conducted targeted glycomic profiling of the Fab and Fc regions of serum IgG to delineate its glycosylation characteristics and provide a comparative reference.

## 2. Results

### 2.1. Overall Work Flow of Glycomic and Glycoproteomic Analyses

**Figure 1:**
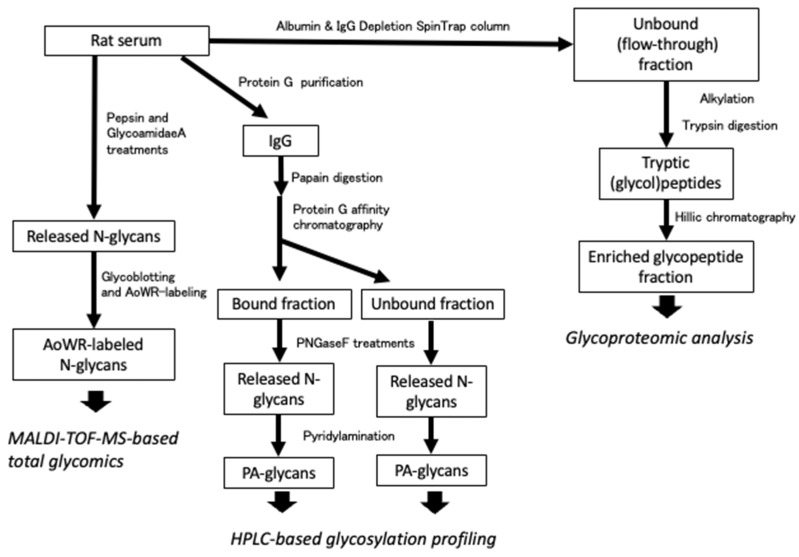
Overview of analytical workflows of rat serum *N*-glycomics and *N*-glycoproteomics. The left panel illustrates the total glycomic workflow. Intact rat serum was subjected to PNGase F digestion to release *N*-glycans, which were labeled with AoWR and analyzed using MALDI-TOF-MS to profile global *N*-glycan compositions. The middle panel shows the IgG-specific glycomic workflow. IgG was purified from serum and digested with papain to generate Fab and Fc fragments, which were separated by protein G affinity chromatography. *N*-glycans were enzymatically released from each fragment by PNGase F, labeled, and analyzed using HPLC mapping for region-specific glycan profiling. The right panel depicts the glycoproteomic workflow. Serum samples were depleted of serum albumin and IgG, digested with trypsin, and glycopeptides were enriched by hydrophilic interaction chromatography (HILIC). LC-MS/MS analysis was performed to identify glycosylation sites and their associated *N*-glycan structures.

Figure 1 illustrates the overall workflow of the glycomic and glycoproteomic analyses conducted in this study. To investigate sex differences and daily variations in the glycome, *N*-glycans released from the whole serum glycoproteins by protein *N*-glycosidase F (PNGase F) treatment were labeled with aminooxy-functionalized tryptophanyl arginine methyl ester (aoWR) and subsequently analyzed using MALDI-TOF-MS [16]. Furthermore, glycoproteomic analysis was performed to identify the proteins carrying these *N*-glycans.

The concentration of albumin in serum is extremely high compared to other serum proteins, which interferes with proteomic analysis. In addition, the *N*-glycopeptides derived from the variable regions of antibodies in the serum are highly diverse in sequence, making their identification challenging. For these reasons, and to ensure high-sensitivity glycoproteomic analysis, serum samples are typically processed to remove albumin and IgG beforehand. This approach was also adopted in this study. Specifically, the rat serum was fractionated to deplete albumin and IgG using a specific affinity column. The flow-through fraction, containing unbound serum glycoproteins, was subjected to tryptic digestion. The resulting tryptic peptides carrying *N*-glycans (*N*-glycopeptides) were enriched using a HILIC column and subsequently analyzed using LC-MS/MS, with identification performed using a database search.

On the other hand, to obtain an IgG fraction, the same serum was subjected to a protein G column. IgG was digested with papain to separate it into Fab and Fc fractions. From each fraction, *N*-glycans were liberated by PNGase F treatment and pyridylaminated for their identification by HPLC mapping technique using the GALAXY database as previously described [17,18,19,20].

### 2.2. Day-to-Day Variations in the N-Glycome of Rat Serum

First, we studied the *N*-glycome of whole serum glycoproteins from four male and four female rats. We employed MALDI-TOF-MS analysis of *N*-glycans released from rat serum glycoproteins by the action of PNGase F, an enzyme that cleaves the bond between the asparagine residues on a protein and the innermost GlcNAc of *N*-glycans attached to that protein. *N*-glycans can be of high mannose, hybrid, or complex type. The latter are branched so that there are bi-antennary, triantennary, or even tetraantennary *N*-glycans. These glycans often end in sialic acid (Sia) residues, which were predominantly Neu5Ac in our observations.

**Figure 2:**
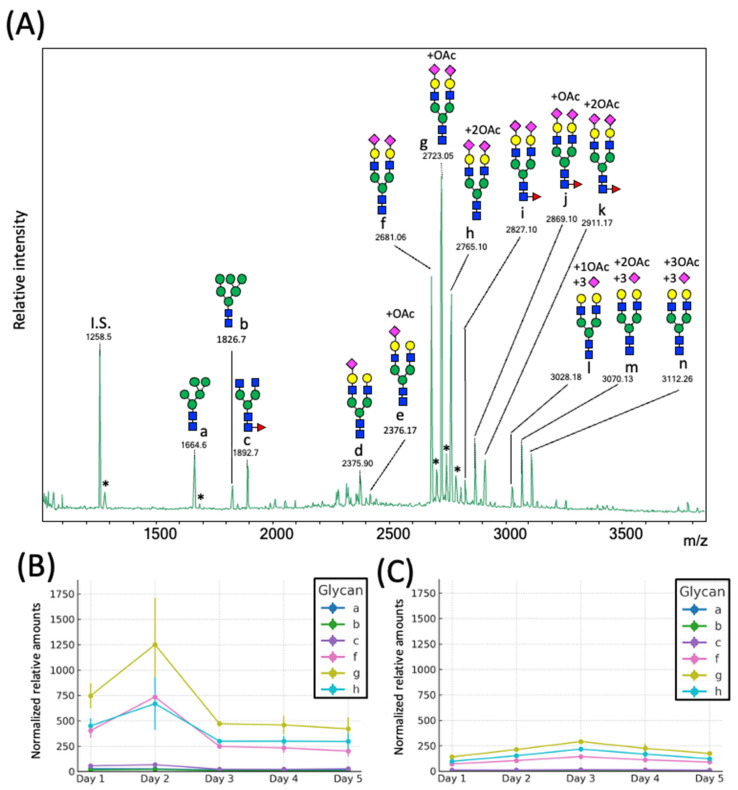
*N*-glycome profiling of total rat serum proteins. (**A**) Representative MALDI-TOF mass spectrum of *N*-glycans released from rat serum glycoproteins. Asterisks mark peaks corresponding to sodium adduct ions of *N*-glycans. Glycan compositions and putative structures were inferred from key diagnostic fragment ions and by referencing established *N*-glycan structures documented in the literature. Mannose (Man), galactose (Gal), fucose (Fuc), *N*-acetylglucosamine (GlcNAc), and *N*-acetylneuraminic acid (Neu5Ac) residues are depicted using the Symbol Nomenclature for Glycans (SNFG) system (https://www.ncbi.nlm.nih.gov/glycans/snfg.html (accessed on 24 July 2025)). (**B**,**C**) Relative amounts of *N*-glycans corresponding to major peaks a, b, c, f, g, and h (Figure 2A), normalized to the internal control, were compared between (**B**) female and (**C**) male rats across five consecutive days. Error bars represent standard deviations (SD) obtained from three technical replicates measured using MALDI-TOF-MS.

We revealed that the predominant *N*-glycans carried by the rat serum glycoproteins are disialo-biantennary complex-type structures, in which one or both Neu5Ac residues could be *O*-acetylated (Figure 2A). The major sialic acid species were Neu5Ac and *O*-acetylated Neu5Ac. In contrast, *N*-glycolylneuraminic acid (Neu5Gc)-containing structures were detected, despite Neu5Gc being commonly found in most non-human mammals [21,22].

The abundance of the disialo-biantennary *N*-glycans was significantly higher in females than in male rats and exhibited substantial interindividual and day-to-day variation. In contrast, the inter-individual variance in *N*-glycan composition in males was small and not prone to day-to-day variations (Figure 2B,C and Figure S1).

### 2.3. Identification of Rat Serum N-Glycoproteins

To investigate which proteins carry the *N*-glycans identified through glycomic analysis, we subsequently conducted glycoproteomic analysis using serum from a single male rat, selected for its stable *N*-glycosylation profile. The tryptic *N*-glycopeptides derived from the albumin/IgG-depleted serum fraction were identified by searching against the SwissProt Rat database for protein sequences and a glycan database containing 132 human *N*-glycan structures modified to reflect rat-specific glycan features. To improve coverage, we additionally searched against a library of *N*-glycan structures obtained from our previous study on the rat serum glycosylation profile [12,13], as well as the 745 glycans included in the Byonic library as *N*-glycan modification.

For peptide-spectrum match (PSM) validation, only spectra with acceptable error probabilities were retained. As a result, a total of 1256 *N*-glycopeptides were confidently identified, corresponding to 147 *N*-glycosylation sites across 87 glycoproteins in the albumin/IgG-depleted serum fraction (Tables S1 and S2).

Through gene ontology (GO) enrichment analysis of the 87 identified glycoproteins (Table S3), we revealed that within the molecular function category, the most significantly enriched term was “endopeptidase inhibitor activity”. The latter was followed by “peptidase inhibitor activity”, “peptidase regulator activity”, and “endopeptidase regulator activity”. These functional categories were commonly associated with 25 glycoproteins, including complement C3, fetuin-B, protein AMBP, T-kininogen 1, and serine protease inhibitor A3K.

**Figure 3:**
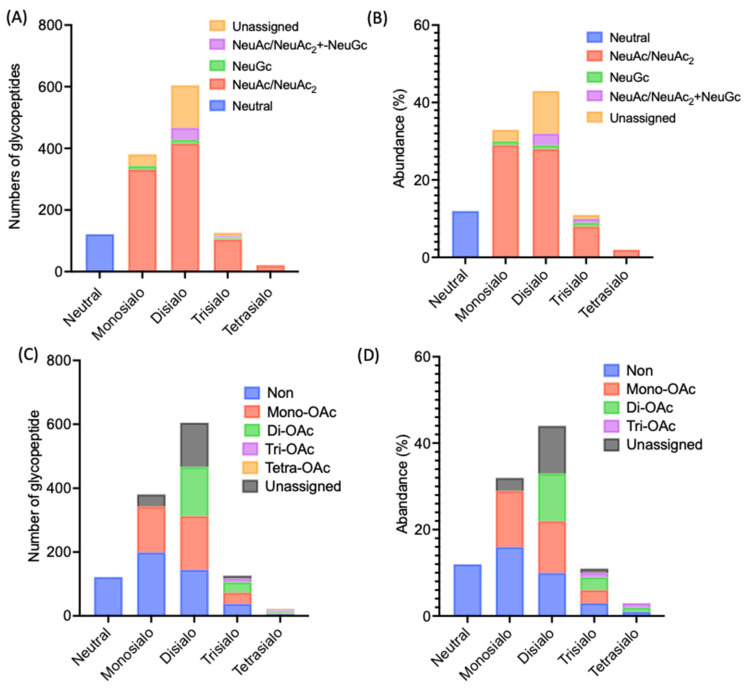
Sialylation and *O*-acetylation status of *N*-glycopeptides derived from *N*-glycoproteins in male rat serum. (**A**,**B**) Distribution of Sia-containing moieties attached to *N*-glycopeptides, shown as (**A**) peptide counts and (**B**) relative abundance. (**C**,**D**) Distribution of *O*-acetylated *N*-glycans in the identified *N*-glycopeptides, also shown as (**C**) peptide counts and (**D**) relative abundance. Glycopeptides were categorized based on their Sia composition into Neu5Ac, *O*-acetylated Neu5Ac (Neu5Ac_2_), and Neu5Gc-containing species. *O*-acetylation was further subclassified by modification type.

Approximately 85% of the glycopeptides identified from the albumin/IgG-depleted serum fraction were sialylated. Most (>75%) of the Sia residues in this fraction were Neu5Ac (Figure 3A,B), and more than half of them were *O*-acetylated (Figure 3C,D), forming Neu5Ac_2_, while Neu5Gc was the least abundant (Figure 3A,B). We employed the extracted ion chromatogram (XIC) data, which display signal intensities for specific *m*/*z* values (corresponding to different *N*-glycopeptides) as a function of retention time, enabling direct comparison of the abundance and retention patterns of glycopeptides bearing Neu5Ac, Neu5Ac_2_, and Neu5Gc residues (Figure S2). Neu5Ac_2_-containing glycans exhibited strong and distinct peaks, whereas signals corresponding to Neu5Gc-derived fragments were markedly lower in intensity.

**Figure 4:**
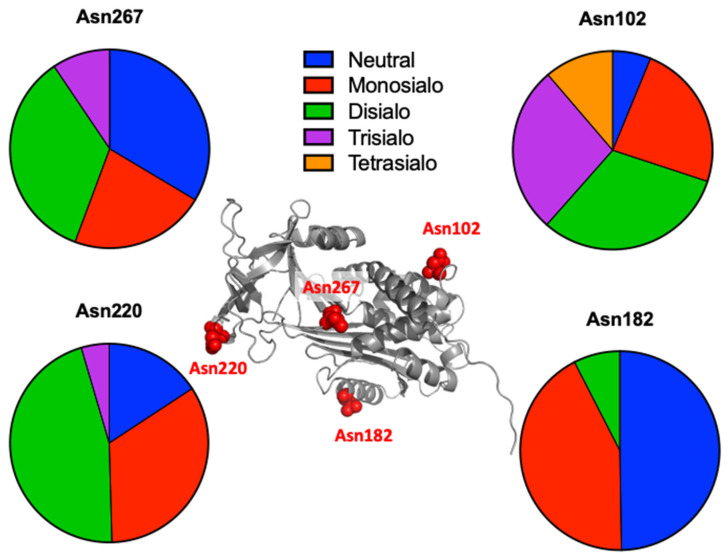
Site-specific *N*-glycosylation of serine protease inhibitor A3K. The 3D structure model of serine protease inhibitor A3K (AlphaFold PDB code AF-P05545-F1-v4) displaying four potential *N*-glycosylation sites (red): Asn102, Asn182, Asn220, and Asn267. The pie charts illustrate the distribution of sialo-*N*-glycans identified at each site.

While glycan profiling revealed diverse *N*-glycans, including mono-, di-, and tri-sialylated structures, bi-antennary disialyl *N*-glycans were the most abundant type in both male and female rats, with their Sia residues often *O*-acetylated. These disialyl *N*-glycans were abundantly observed in the glycomic analyses of rat whole serum (Figure 2) and were found in several major glycoproteins, such as hemopexin, alpha-1-macroglobulin, beta-2-glycoprotein 1, and complement component C4 (Table S1). We then examined the site-specific *N*-glycoproteomic profile of rat serum. As an illustration, serine protease inhibitor A3K—a major liver-derived glycoprotein—possesses four *N*-glycosylation sites: Asn102, Asn182, Asn220, and Asn267 (Figure 4). Each of them carries a sialylated complex-type *N*-glycan, which can be *O*-acetylated or not. Notably, the predominant structure shared across these four sites is the aforementioned biantennary complex type *N*-glycan structure bearing terminal Neu5Ac and one *O*-acetylated Neu5Ac. Among these sites, Asn102 is characterized by increased branching of *N*-glycans, the majority (23.2%) of which contain one or two *O*-acetyl groups. This site-specificity is likely associated with A3K’s three-dimensional structure, as the accessibility of glycosyltransferases is a crucial factor in *N*-glycan maturation [23,24].

**Figure 5:**
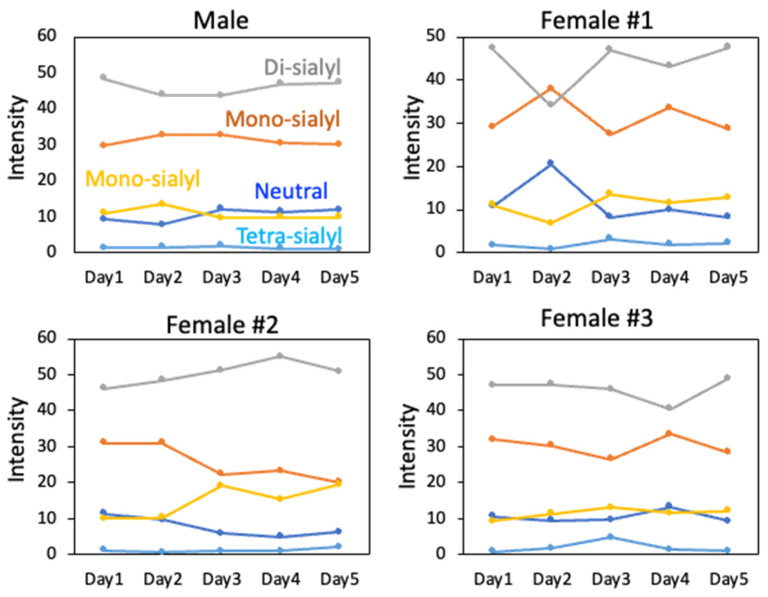
Temporal changes in sialylation of *N*-glycopeptides in rat serum. Points in graphs represent combined intensities of all *N*-glycopeptides carrying terminal Sia residues in the following moieties: neutral (no Sia); monosialo, disialo, trisialo, and tetrasialo *N*-glycopeptides. The *N*-glycopeptides were derived from rat serum (one male rat and three female rats) obtained over five consecutive days.

We extended our glycoproteomic analysis to female rats. Daily samples were collected over five consecutive days from three female rats, together with one male control rat, which were then subjected to glycoproteomic analysis. While some quantitative variations were observed, the identified glycoproteins in the female rats were consistent with those found in the male control (Table S2), indicating that the same glycoproteins were present, albeit at different levels. Notably, unlike the male control, which exhibited minimal day-to-day variation in the sialylation level of whole glycopeptides, the female rats showed significant fluctuations over the five-day period (Figure 5), consistent with the glycomics data described earlier (Figure 2).

### 2.4. N-Glycosylation Profiles of Rat Serum IgG

Here, we also investigated the glycosylation profile of the IgG fraction removed from serum in glycoproteomic analysis. The Fc region of IgG bears two conserved *N*-glycosylation sites present across various mammalian species [25]. On the other hand, a subset of IgG molecules may carry *N*-glycosylation sites in the variable domains of the Fab region. The latter are challenging to identify using LC-MS/MS due to the lack of corresponding sequence data in peptide-matching libraries.

To investigate the *N*-glycosylation status of Fab and Fc fragments of rat IgG, the protein was purified from male rat serum, enzymatically cleaved at the hinge region by the action of papain. The fragments were separated on the immobilized protein G, recovered, and subjected to PNGase F. The enzyme liberated *N*-glycans from the Fab and Fc fragments, which were then fluorescently labeled so that they could be mapped through HPLC.

**Figure 6:**
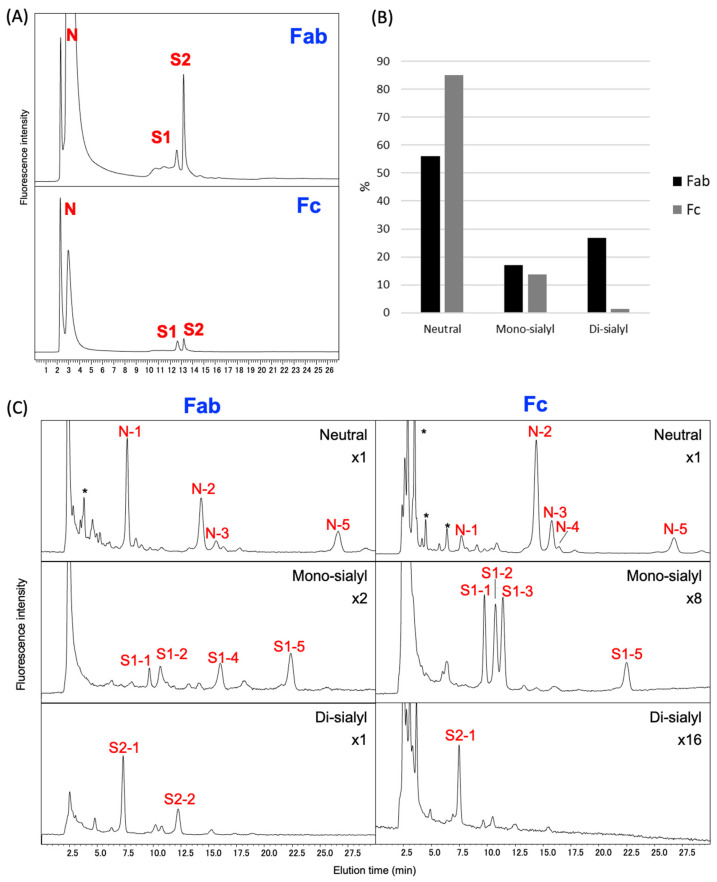
Comparative glycan profiling of Fab and Fc fragments of rat serum IgG. (**A**) HPLC profiles of *N*-glycan using DEAE anion-exchange chromatography. *N*-glycans released from Fab and Fc fragments were separated into three fractions based on their charge: N (neutral), S1 (mono-sialylated), and S2 (di-sialylated). (**B**) Relative abundance of N-, S1-, and S2-glycans in Fab and Fc fragments, as determined by the signal intensity of each ODS fraction. (**C**) HPLC profiles of ODS column of the fraction separated by DEAE column (N, S1, and S2). The *N*-glycans structure was identified using the HPLC mapping method in conjunction with MALDI-TOF-MS analysis. Peaks unassignable to *N*-glycans are indicated by asterisks.

We found that both Fab and Fc fragments carry *N*-glycans terminating in Sia residues, exclusively of the Neu5Gc type. (Figure 6 and Table 1). The Fab fragment of rat IgG exhibited a higher degree of sialylation compared to the Fc fragment, predominantly featuring disialo-*N*-glycan structures. Quantitatively, sialylated *N*-glycans accounted for approximately 44% of the total glycan pool in Fab, whereas only about 15% were observed in Fc, highlighting a threefold difference in sialylation levels. These findings are consistent with previous reports on IgG *N*-glycosylation patterns in other species, which also demonstrated higher levels of sialylation in the Fab region compared to the Fc region [26,27,28]. Notably, virtually no *N*-glycans containing *O*-acetylated Sia residues were detected in either fragment.

It is worth noting that during the immunoblot analysis, we observed that the Fc fragment did not bind to protein G, whereas the Fab fragment did (Figure S3). Proteomic analysis of the protein G-bound and flow-through fractions supported this observation. In the protein G-bound fraction, we detected solely the κ (kappa) light chain of IgG (Table S4), a hallmark of Fab, although it is the Fc fragment that is supposed to bind to protein G. In contrast, proteomic analysis of the flow-through fraction of the protein G-column, where we expected Fab, supported the presence of the Fc regions of IgG2b, IgG2a, IgG1, and IgG2c molecules. Therefore, the Fc fragments derived from the rat serum IgG showed no affinity for protein G. To sum up, in rats, the Fc fragment does not bind to protein G, whereas the Fab fragment does.

## 3. Discussion

In this study, we systematically characterized the *N*-glycosylation landscape of rat serum glycoproteins using samples from genetically defined animals of controlled age and sex. A distinguishing feature of our approach is the separate analysis of IgG and non-IgG fractions, enabling cell-type-resolved insights. The findings reveal a distinct sialylation pattern: Neu5Gc-only sialylation in B-cell-derived IgG and Neu5Ac or its *O*-acetylated derivatives in liver-derived glycoproteins, such as A3K and C3.

Importantly, our results are consistent with previous studies and do not contradict existing glycomic and glycoproteomic data [12,13,15]. Earlier reports provided a comprehensive serum-wide glycomic profile of rats, revealing the co-occurrence of Neu5Ac and NeuGc residues, as well as extensive *O*-acetylation on multi-sialylated *N*-glycans, particularly those containing Neu5Ac. However, these studies did not differentiate between IgG and other serum glycoprotein species. Our findings refine this picture by demonstrating that *O*-acetylation is absent in rat IgG, which exclusively carries Neu5Gc, thus confirming structural observations while adding cell-type specificity. This is consistent with the high-throughput IgG glycosylation profiles reported by Habazin et al. [14], who identified Neu5Gc-containing glycoforms in rat IgG under stress conditions; however, their study did not address *O*-acetylation or provide a structural resolution of the Neu5Gc species.

This cell-type specificity of sialylation patterns appears to be species-dependent. In humans, both liver-derived glycoproteins and B-cell-produced IgG contain Neu5Ac as the sole sialic acid, and *O*-acetylation is negligible [15,29,30]. In mice, liver glycoproteins are rich in Neu5Gc, while B-cell-produced IgG tends to carry Neu5Ac [15,31]. In both species, the *O*-acetylation of Neu5Ac is essentially absent [12,15]. These features contrast sharply with rats, where liver glycoproteins exhibit extensive *O*-acetylation of Neu5Ac, and IgG exclusively carries Neu5Gc without *O*-acetylation.

**Figure 7:**
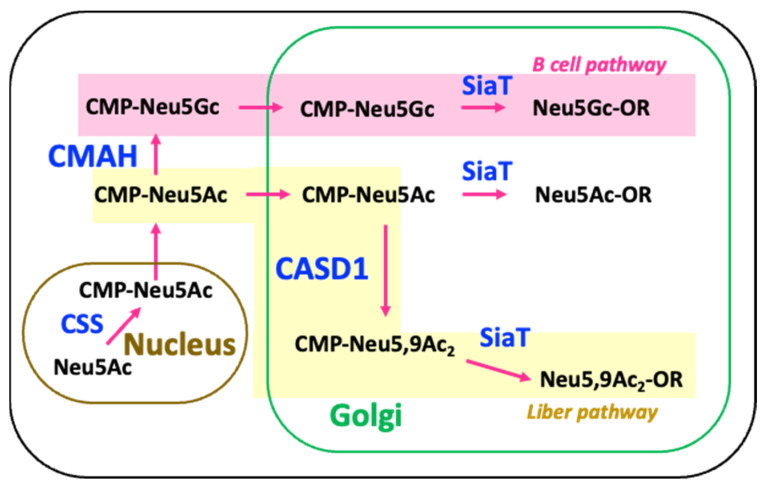
Schematic representation of intracellular pathways for CMP-sialic acid modification and utilization in rat liver and B cells. CMP-Neu5Ac, synthesized in the nucleus via the Sia biosynthetic enzyme CSS, serves as a common precursor for further modifications in the cytoplasm and Golgi. CASD1 catalyzes *O*-acetylation at the C9 position of CMP-Neu5Ac in the Golgi, producing CMP-Neu5,9Ac_2_, which is then transferred to glycoproteins by sialyltransferases (SiaT), resulting in Neu5,9Ac_2_-modified glycoconjugates. In contrast, CMAH hydroxylates CMP-Neu5Ac into CMP-Neu5Gc, leading to the incorporation of Neu5Gc into glycoconjugates via the same SiaT-dependent mechanism.

In rat serum, the *O*-acetylation of *N*-acetylneuraminic acid (Neu5Ac) predominantly occurs at the C9 position [13]. This modification is initiated in the Golgi apparatus, where the donor substrate CMP-Neu5,9Ac_2_ is synthesized from CMP-Neu5Ac via C9-specific *O*-acetylation catalyzed by CASD1 (Cas1 domain-containing protein 1) (Figure 7) [32]. The unusually high abundance of *O*-acetylated Neu5Ac in rat liver, relative to human and mouse, is likely attributable to enhanced expression or enzymatic activity of CASD1.

On the other hand, it seems that in rat B cells, CMAH (cytidine monophosphate-*N*-acetylneuraminic acid hydroxylase), which converts CMP-Neu5Ac to CMP-Neu5Gc, is highly active; hence, the high content of Neu5Gc on rat serum IgG (Figure 7). In humans, the *CMAH* gene is either deleted or non-functional [33], resulting in a general lack of Neu5Gc moieties on the termini of *N*-glycans. Similarly, in mice germinal center B cells, the expression of the CMAH enzyme is suppressed [34], so the *N*-glycans of mice IgG are primarily characterized by Neu5Ac. As a result, the sialylated glycans of mouse and human IgG are primarily characterized by Neu5Ac. In contrast, mouse liver expresses functional CMAH, leading to the biosynthesis of Neu5Gc-rich glycoproteins in hepatic tissues [15,35].

These interspecies differences in sialic acid biosynthesis may reflect divergent evolutionary pressures and distinct immunological roles. In humans, Neu5Gc is immunogenic and has been implicated in xeno-autoantibody formation and inflammation when incorporated from dietary sources [36]. By contrast, the endogenous presence of Neu5Gc in rat serum IgG may represent a tolerated or functionally integrated glycoform. This divergence raises intriguing questions about the role of terminal sialic acid identity—Neu5Ac vs. Neu5Gc—in IgG-mediated immune modulation across species [37].

Moreover, terminal sialic acid structures have been shown to influence the circulatory half-life of glycoproteins [25,38]. Neu5Gc-rich glycoforms may be differentially recognized by hepatic asialoglycoprotein receptors or sialic acid-binding immunoglobulin-type lectins (Siglecs), potentially altering the clearance rate [37]. Although the precise impact of Neu5Gc on IgG pharmacokinetics remains to be elucidated, the predominance of Neu5Gc, particularly in Fab glycans of rat IgG, observed here raises the possibility of species-specific turnover dynamics and immune homeostasis.

Kinoshita et al. reported that the *N*-glycosylation of serum glycoproteins in male rats is age-dependent and alters upon food restriction [13]. Specifically, the abundance of 9-*O*-acetylated disialo-biantennary *N*-glycans significantly increased with aging. Moreover, age-related *O*-acetylation of Sia residues was suppressed by a high-fat diet [13]. The total amount of disialo-biantennary *N*-glycans on all *N*-glycoproteins in rat serum grew with age [10]. In the study presented herein, we revealed that the proportion of sialo-*N*-glycans is higher in females than in males. In addition, the content of these *N*-glycans fluctuates daily on a shorter timescale than age-related changes. A marked increase in disialo-biantennary *N*-glycans with specifically Neu5Ac or Neu5,9Ac_2_ was detected. Based on the types of Sia residues identified, these fluctuations in sialo-*N*-glycan abundance can be attributed primarily to non-IgG glycoproteins present in rat serum.

Through an *N*-glycoproteomic analysis, we identified several serum proteins that carried Neu5Ac or Neu5,9Ac_2_, including T-kininogen 1, alpha-1-macroglobulin, complement C4, and serine protease inhibitor A3K, which were elevated in the serum of female rats. As the aforementioned proteins are made in the liver, their increased expression is likely influenced by sex hormones, particularly estrogens [39,40]. Previous studies in rats have shown that sex-biased expression of hepatic (glyco)proteins can result from transcriptional regulation by the growth hormone–STAT5 signaling pathway [41]. Consistent with this, our findings—based on liver-derived glycoproteins from female and male rats—indicate that sex-dependent differences in the abundance of specific *N*-glycan structures, particularly terminal sialylation, are closely associated with the expression levels of glycoproteins in female rat serum. These patterns are likely influenced by hormonal fluctuations linked to the estrous cycle and circadian rhythms.

Taken together, our findings refine the current understanding of cell-type and sex-specific *N*-glycan distributions in rat serum glycoproteins, particularly in relation to sialylation and *O*-acetylation profiles. Although this study does not directly explore the enzymatic pathways underlying glycan biosynthesis, it provides a quantitative framework for assessing physiological variability in the serum *N*-glycome and reinforces the utility of rat models in glycomic and glycoproteomic research. The observed daily fluctuations in specific sialylation of *N*-glycans derived from serum glycoproteins detected in female rats imply a link between *N*-glycosylation and hormonal cycles, thereby shedding light on the dynamic regulation of *N*-glycoproteins under different pathophysiological conditions. These insights pave the way for future research into the functional roles of *N*-glycosylation patterns in disease pathogenesis, biomarker discovery, and the advancement of glycoengineering strategies for therapeutic innovation.

Each pathway leads to the generation of structurally distinct sialylated glycans—Neu5,9Ac_2_ in liver-derived proteins and Neu5Gc in B cell-derived proteins—highlighting cell-type specific regulation of Sia diversification.

## 4. Materials and Methods

### 4.1. Rat Sera

Animal experiments were approved by the Ethics Committee of Nagoya City University (R2-P-03), and all procedures were conducted in accordance with the guidelines of the National Institute of Health Sciences of Japan. Eighteen-month-old male and female Wistar/ST rats were housed in standard cages (two per cage) in an animal care facility. The rats were kept at a constant temperature (22 °C) under a 12 h light/dark cycle.

Approximately 500 μL of blood was collected from the tail vein of a conscious rat restrained in a fixation device using a 23-gauge winged needle (Terumo, Tokyo, Japan). The blood was allowed to clot at room temperature in a 1.5 mL tube (BIO-BIK, Osaka, Japan), centrifuged at 3000× *g* for 10 min, and the supernatant (serum) was collected. For female rats and control male rats, considering that the estrous cycle in rodents lasts 4–5 days, blood sampling was performed at the same time each day for 5 consecutive days following a 4-day acclimation period. On the final day, terminal blood collection was performed to obtain the entire blood volume for serum preparation.

### 4.2. Analysis of Day-to-Day Variations in the N-Glycome of Rat Serum

Glycomic analysis was carried out following the method described in the previous study [16], using serum collected from four male and four female rats over five consecutive days. *N*-glycans were released from 50 µL of rat serum by treatment with PNGase F (New England Biolabs, Ipswich, MA, USA) following standard denaturation and reduction procedures. Serum proteins were denatured at 95 °C for 5 min in the presence of 1% SDS and 50 mM DTT, and subsequently alkylated with 100 mM IAA at room temperature for 30 min in the dark. After neutralization of SDS with 1% NP-40, the samples were incubated with PNGase F at 37 °C overnight to enzymatically release *N*-glycans.

The released glycans were oxidized with sodium periodate and purified using the GlycoBlot™ system (Sumitomo Bakelite, Tokyo, Japan), which enables selective capture of oxidized glycans onto hydrazide-functionalized beads. After washing away the unbound components, the immobilized glycans were directly labeled on the beads with aminooxy-functionalized tryptophanyl derivative (aoWR). The labeling reaction was carried out in acetonitrile containing 2% acetic acid at 80 °C by imine exchange reaction. The aoWR-labeled glycans were then eluted from the beads and desalted using a HILIC μElution plate.

For MALDI-TOF-MS analysis, the aoWR-labeled glycans were then eluted from the beads and desalted using reversed-phase solid-phase extraction.

For MALDI-TOF-MS analysis, the aoWR-labeled glycans were mixed with an equal volume of 2,5-dihydroxybenzoic acid (20 mg/mL in 50% acetonitrile with 0.1% trifluoroacetic acid), and 1 µL of the mixture was spotted onto a MALDI target plate and allowed to air-dry. Mass spectra were acquired using an Autoflex Speed instrument (Bruker Daltonics, Billerica, MA, USA) in positive-ion reflector mode, with 5000 laser shots accumulated per spot. The instrument settings included a laser intensity of 50–78%, resulting in a total of 5000 shots for reflector positive mode MS. The MS survey and data acquisition were performed manually. The acquired mass spectra were processed using flexAnalysis 3.4 software (Bruker Daltonics, Billerica, MA, USA).

### 4.3. Glycoproteomic Analysis of Serum Glycoproteins Other Than IgG

#### 4.3.1. Sample Preparation

Glycoproteomic analysis was performed with modifications to a previously described method [42], using serum collected over five consecutive days from one male and four female rats. Albumin and IgG were removed from serum using the Albumin & IgG Depletion SpinTrap column (Cytiva, Marlborough, MA, USA) following the manufacturer’s protocol. Briefly, 50 µL rat plasma was diluted in 20 mM sodium phosphate buffer, pH 7.4, containing 150 mM NaCl to a final volume of 100 µL. The diluted serum was loaded onto a pre-equilibrated albumin and IgG depletion SpinTrap column, and the flow-through (unbound) fraction devoid of serum albumin and IgG was subjected to *N*-glycoproteomic analysis. The protein fraction was dried using vacuum centrifugation. The dried samples were resuspended in 50 µL of PTS buffer (100 mM Tris-HCl, pH 8.5, 12 mM sodium deoxycholate, and 12 mM sodium lauroylsarcosinate). Proteins (~100 µg) from 10 µL of rat serum were reduced by adding DTT to a final concentration of 4 mM, followed by incubation for 30 min at 30 °C. Alkylation was performed by adding IAA to a final concentration of 8 mM and incubating the mixture in the dark at room temperature for 30 min. To quench the reaction, DTT was added to a final concentration of 8 mM. Proteins were digested overnight at 37 °C with a Trypsin/LysC mix (1:50, *w*/*w*, spec-grade, Promega, Madison, WI, USA). Glycopeptides were enriched using a hydrophilic interaction liquid chromatography (HILIC) enrichment method. Tryptic peptides were mixed with 50 mg of SigmaCell Cellulose (Sigma-Aldrich, St. Louis, MO, USA) in 98% acetonitrile and 1% trifluoroacetic acid (TFA). The mixtures were rotated at 1500 rpm at room temperature for 30 min. SigmaCell cellulose was washed twice for 5 min each with 1% TFA and 80% acetonitrile. Glycopeptides bound to the cellulose were eluted twice with 200 µL of 0.1% TFA and 20% acetonitrile.

#### 4.3.2. LC-MS/MS Measurements

Purified glycopeptides were analyzed by LC-MS/MS using an Orbitrap Exploris 240 mass spectrometer coupled to an UltiMate 3000 RSLCnano system (Thermo Fisher Scientific, Waltham, MA, USA). Peptide mixtures were loaded onto an NTCC-360/75-3-155 column (Niko Technos Co., Ltd., Tokyo, Japan) and separated at a flow rate of 300 nL/min using a gradient of 1% to 35% solvent B (0.1% formic acid in acetonitrile) over 35 min. Solvent A consisted of 0.1% formic acid in water.

MS and MS/MS data acquisition were performed in HCD product ion-triggered CID mode. The top-speed acquisition mode was set with a 3-second cycle time. For FTMS, the scan range was set to *m*/*z* 400–1800 with a resolution of 60,000, an AGC target of 300%, and a maximum injection time of 50 ms. Monoisotopic precursor selection was enabled, and charge states from 2 to 8 were included. Dynamic exclusion was applied after a single occurrence, with a 15-second exclusion time and a 10 ppm mass tolerance. For FTMSn (HCD), the isolation mode used a quadrupole with an isolation window of 1.6 *m*/*z*. Collision energies of 20%, 30%, and 40% were applied, with a resolution of 15,000, an AGC target of 200%, and a maximum injection time of 100 ms.

#### 4.3.3. Data Analysis

A database search was performed using Proteome Discoverer 2.5 with Sequest HT (Thermo Fisher Scientific, Waltham, MA, USA) against the SwissProt Rat database. Peptide sequencing was conducted on fully trypsin-digested proteins, allowing a maximum of two missed cleavages. The mass tolerance was set to 10 ppm for precursor ions and 0.02 Da for fragment ions. Carbamidomethylation of cysteine was specified as a static modification, while dynamic modifications included the oxidation of methionine, acetylation, methionine loss, and methionine loss combined with acetylation at the N-terminus.

Qualitative and quantitative analyses of enriched glycopeptides were performed using Byonic software version 4.3 (Protein Metrics Inc., Cupertino, CA, USA) and Proteome Discoverer 2.5. Searches were conducted against the SwissProt Rat database and a glycan database containing 132 human *N*-glycans modified for rat glycan structures. Glycopeptide sequencing was configured for trypsin digestion with a maximum of two missed cleavages, a 10 ppm mass tolerance for precursor ions, and a 0.5 Da mass tolerance for product ions. Carbamidomethylation of cysteine was included as a static modification. The results were manually evaluated, and peptides lacking an *N*-glycosylation consensus sequence were excluded.

### 4.4. N-Glycosylation Profiling of Rat Serum IgG

#### 4.4.1. Sample Preparation

For IgG preparation, 1 mL of pooled rat serum (prepared by combining the remaining serum from male rats used for glycomics analysis) was diluted with 20 mM sodium phosphate buffer, pH 7.4, containing 150 mM NaCl and loaded onto a column packed with protein G immobilized on Sepharose (Cytiva, Marlborough, MA, USA). The purified IgG fraction was incubated with papain at an enzyme/substrate ratio of 1/100 (*w*/*w*) in 75 mM sodium phosphate buffer, pH 7.0, containing 1 mM cysteine, 75 mM NaCl, and 2 mM EDTA for 17 h at 37 °C. Papain activity was quenched by adding 33 mM N-ethylmaleimide to the reaction mixture. The reaction mixture was then applied to a protein G column equilibrated with 10 mM sodium phosphate buffer, pH 7.4, containing 150 mM NaCl for separation into bound and unbound fractions.

#### 4.4.2. Identification of Fab and Fc Fractions

The Fc and Fab fractions were identified using immunoblotting analysis and further confirmed using LC-MS/MS analysis.

For immunoblotting, the bound and unbound fractions obtained from protein G affinity chromatography were resolved by sodium dodecyl sulfate polyacrylamide gel electrophoresis (SDS-PAGE) on 12% polyacrylamide gels under reducing conditions. After electrophoresis, proteins were transferred onto PVDF membranes (Immobilon-P, Millipore, Burlington, MA, USA) using a semi-dry blotting system. The membranes were blocked with Blocking One (Nacalai Tesque, Tokyo, Japan) in TBS-T buffer (20 mM Tris-HCl, 150 mM NaCl, 0.1% Tween-20, pH 7.5) for 1 h at room temperature. Subsequently, the membranes were incubated overnight at 4 °C using two horseradish peroxidase (HRP)-conjugated antibodies produced in goats: an antibody against the rat IgG light chain (Cell Signaling, Danvers, MA, USA, 98164) and anti-rat IgG antibody (Cytiva, Marlborough, MA, USA, NA935). After three washes with TBS-T, chemiluminescent signals were developed using an ECL detection reagent (Millipore, Burlington, MA, USA) and visualized with an AI600 imaging system (GE Healthcare, Chicago, IL, USA). Chemiluminescent signals were developed using an ECL detection reagent (Millipore, Burlington, MA, USA) and visualized using an AI600 imaging system (GE Healthcare, Chicago, IL, USA).

The identity of the Fab and Fc fractions was further confirmed through LC-MS/MS-based label-free protein quantification. Proteins in the bound and unbound fractions from protein G affinity chromatography were reduced with 10 mM dithiothreitol (DTT) at 56 °C for 30 min and alkylated with 20 mM iodoacetamide (IAA) at room temperature in the dark for 30 min. The samples were then digested with Trypsin/Lys-C Mix (Promega, Madison, WI, USA) at a 1:50 enzyme-to-substrate ratio and incubated overnight at 37 °C. The resulting peptides were acidified with 0.1% formic acid, desalted using a mono-spin column (GL Science, Tokyo, Japan), and subjected to LC-MS/MS analysis. The peptides were analyzed using a Vanquish Neo UHPLC system equipped with a C18 column (Nikkyo Technos, Tokyo, Japan, NTCC-360/75-3-125), a FAIMS Pro Duo interface, and an Orbitrap Exploris 240 mass spectrometry (Thermo Fisher Scientific, Waltham, MA, USA). Peptide separation was performed using a gradient of solvent A (0.1% formic acid in water) and solvent B (80% acetonitrile with 0.1% formic acid), starting from 6% to 31% solvent B over 15 min, followed by a gradient from 31% to 50% over 5 min at a flow rate of 300 nL/min. Compensation voltages of −45 and −65 V were applied in FAIMS with a total carrier gas flow of 1.2 L/min. MS and MS/MS spectra were acquired in full MS/data-dependent MS2 mode. The full MS scan range was set to 350–1500 *m*/*z* at a resolution of 60,000, and MS2 spectra were acquired with a 1.6 *m*/*z* isolation window at a resolution of 15,000.

Raw proteomic data were analyzed using the SEQUEST algorithm in Proteome Discoverer 3.1 SP1, searching against the UniProt *Rattus norvegicus* FASTA database (UP000002494, downloaded October 2024) and a contaminant database [43]. Carbamidomethylation of cysteine was set as a static modification. Dynamic modifications included oxidation of methionine; deamidation of asparagine and glutamine; N-terminal glutamine to pyroglutamate; N-terminal acetylation; methionine loss at the N-terminus; and combined N-terminal methionine loss and acetylation. Peptide-spectrum matches were filtered using a strict target false discovery rate (FDR) of 0.01 and a relaxed target FDR of 0.05 in a Target Decoy PSM Validator. The Minora Feature Detector node was used to detect chromatographic features for label-free MS1 quantification.

#### 4.4.3. Identification of PA-Glycans Derived from Rat Serum IgG

All experimental procedures for glycan profiling, including the preparation of PA-glycans, chromatographic conditions, and matrix-assisted laser desorption/ionization time-of-flight mass spectrometry (MALDI-TOF-MS), were performed as previously described [17,18,19,20]. Briefly, the Fc and Fab fragments were resuspended in citrate-phosphate buffer (pH 4), and *N*-glycans were enzymatically released using glycoamidase A. The resulting glycan mixture was applied to a graphite carbon column (GL-Pak Carbograph, 150 mg/3 mL; GL Sciences, Tokyo, Japan) for initial purification. The released *N*-glycans were then labeled with 2-aminopyridine, and the resulting pyridylaminated (PA) glycans were further purified using a cellulose column.

The purified PA-glycan mixture was fractionated by reverse-phase high-performance liquid chromatography (HPLC) using a Shim-pack HRC octadecyl silica (ODS) column (6.0 mm I.D. × 150 mm; Shimadzu, Kyoto, Japan). Each fraction was mixed with a matrix solution containing 10 mg/mL 2,5-dihydroxybenzoic acid and analyzed using MALDI-TOF-MS on an Autoflex Speed instrument (Bruker Daltonics, Billerica, MA, USA) in positive-ion reflector mode. Acquired mass spectra were processed using flexAnalysis software version 3.4 (Bruker Daltonics, Billerica, MA, USA).

## Figures and Tables

**Table 1:** *N*-glycans cleaved from Fab and Fc fragments derived from rat serum IgG.

Peak	GU (ODS) *^a^*	Observed Mass Value *^b^*	Structure	Relative Quantity *^c^* (mol %)	
				Fab	Fc
N-1	8.8	- *^f^*	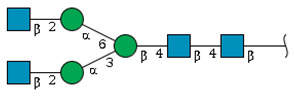	22.8	4.5
N-2	12.0	-	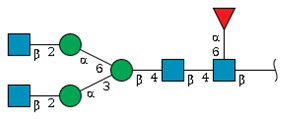	17.7	50.2
N-3	12.6	-	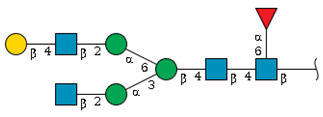	2.8	14.4
N-4	12.8	-	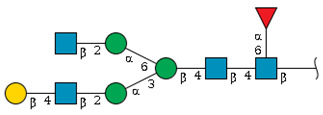	-	2.1
N-5	16.7	-	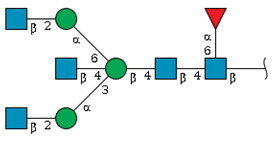	9.4	9.9
N-others *^e^*				3.4	3.7
S1-1	10.0	2054.6	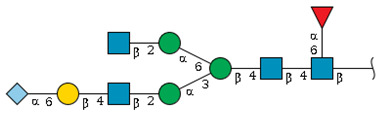	1.6	3.0
S1-2	10.5	2216.7	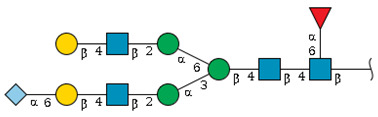	2.3	3.9
S1-3	10.8	-	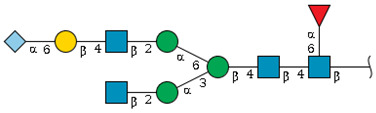	-	4.3
S1-4 *^d^*	12.7	2257.7	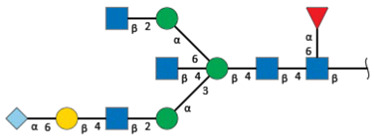	5.0	-
S1-5 *^d^*	15.1	2545.8	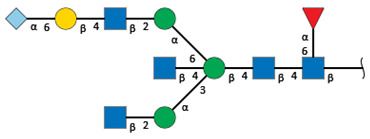	6.5	1.8
S1-others *^e^*				1.6	0.7
S2-1	8.7	2545.8	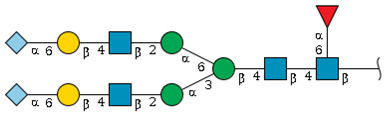	18.2	1.4
S2-1 *^d^*	11.2	2748.9	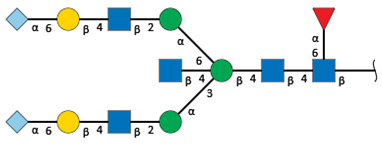	7.2	-
S2-others *^e^*				1.5	0.1
Total				100	100

*^a^* Units of glucose (GU) were calculated from the elution times of the peaks obtained from the ODS column in Figure 6C. *^b^* Average masses were determined from the observed *m*/*z* values corresponding to the mono-sialylated [M−H+2Na]^+^ and di-sialylated [M−2H+3Na]^+^ ions of PA-labeled oligosaccharides analyzed using MALDI-TOF-MS. The molar percentage of individual glycans within each peak was calculated based on the relative peak intensities in the MALDI-TOF-MS spectra. *^c^* The ratio (mol %) was calculated from the peak area in Figure 6C by comparison with the total *N*-glycan content derived from IgG-Fab and Fc fragments. *^d^* The structure was predicted based on unit contribution, which represents the impact of individual monosaccharide units on HPLC elution time, as reported in the GALAXY database. *^e^* Total relative abundance of glycans, with individual proportions below 1.5% for Fab fragments and 1.2% for Fc fragments. *^f^* Not examined.

## Data Availability

The data presented in this study are available upon request from the corresponding authors. The LC data, along with the subsequent Proteomics LC-MS data, MALDI-TOF-MS data, and the MS-based glycoproteomics data, have been deposited in JPOST (Accession No. JPST003882), GlycoPOST (Accession No. GPST000601), and JPOST (Accession No. JPST003883), respectively.

## Supplementary Materials

The following supporting information can be downloaded at: https://www.mdpi.com/article/10.3390/ijms26157266/s1.

## Abbreviations

The following abbreviations are used in this manuscript:

AGC	Automatic gain control
CASD1	Cas1 domain-containing protein 1
CID	Collision-induced dissociation
CMAH	CMP-*N*-acetylneuraminic acid hydroxylase
CSS	CMP-sialic acid synthetase
DEAE	Diethylaminoethyl (anion-exchange chromatography)
DTT	Dithiothreitol
Fab	Fragment antigen-binding
FAIMS	Field asymmetric ion mobility spectrometry
Fc	Fragment crystallizable
GO	Gene ontology
HCD	Higher-energy collisional dissociation
HPLC	High-performance liquid chromatography
HRP	Horseradish peroxidase
IAA	Iodoacetamide
IgG	Immunoglobulin G
LC-MS/MS	Liquid chromatography–tandem mass spectrometry
MALDI-TOF-MS	Matrix-assisted laser desorption/ionization time-of-flight mass spectrometry
MS	Mass spectrometry
Neu5Ac	*N*-acetylneuraminic acid
Neu5,9Ac_2_	*O*-acetylated *N*-acetylneuraminic acid
Neu5Gc	*N*-glycolylneuraminic acid
ODS	Octadecylsilyl (C18 reverse phase column)
PNGase F	Peptide-*N*-Glycosidase F
PSM	Peptide spectrum match
XIC	Extracted ion chromatogram
