# Supplementary material for: Temporal and Sex-Dependent N-Glycosylation Dynamics in Rat Serum
Source: Int J Mol Sci. 2025 Jul 27;26(15):7266. doi: 10.3390/ijms26157266 (PMC12347175; doi:10.3390/ijms26157266)
Supplement: Supplementary file 1 [file ijms-26-07266-s001.zip › Figures S1, S2, S3.pdf]

Figure S1

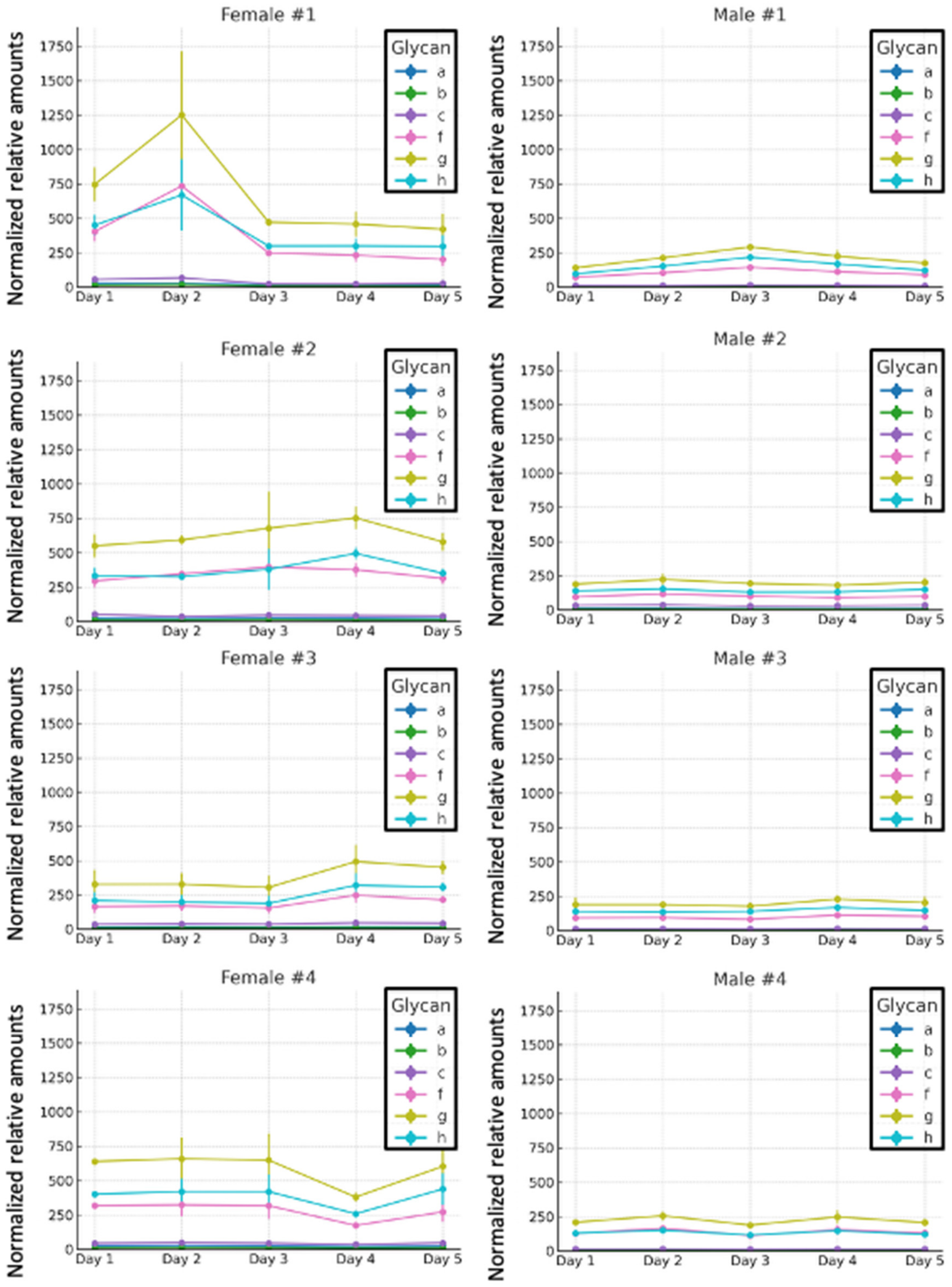

Figure S1. Glycomic profiling of serum *N*-glycans in male and female rats. Relative amounts of N-glycans corresponding to peaks a, b, c, f, g, and h (Figure 2A), normalized to the internal control, were compared between four female and four male

rats over five consecutive days. Error bars represent standard deviations (SD) calculated from three technical replicates measured by MALDI-TOF-MS.

Figure S2

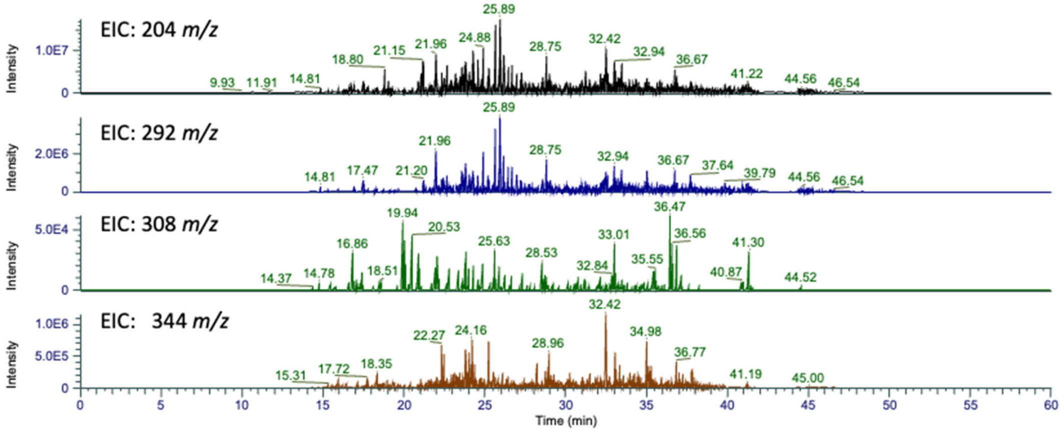

Figure S2. Extracted ion chromatograms of glycopeptides derived from rat serum. The chromatograms display four distinct *m/z* ranges corresponding to diagnostic ions: (204 *m/z*) 204.0847–204.0887 (HexNAc), (292 *m/z*) 292.0998–292.1056 (NeuAc), (308 *m/z*) 308.0945–308.1007 (NeuGc), and (344 *m/z*) 334.1100–334.1166 ($NeuAc_2$). LC-MS analysis of the diagnostic ion associated with NeuGc (308 *m/z*) revealed minimal peak detection.

Figure S3

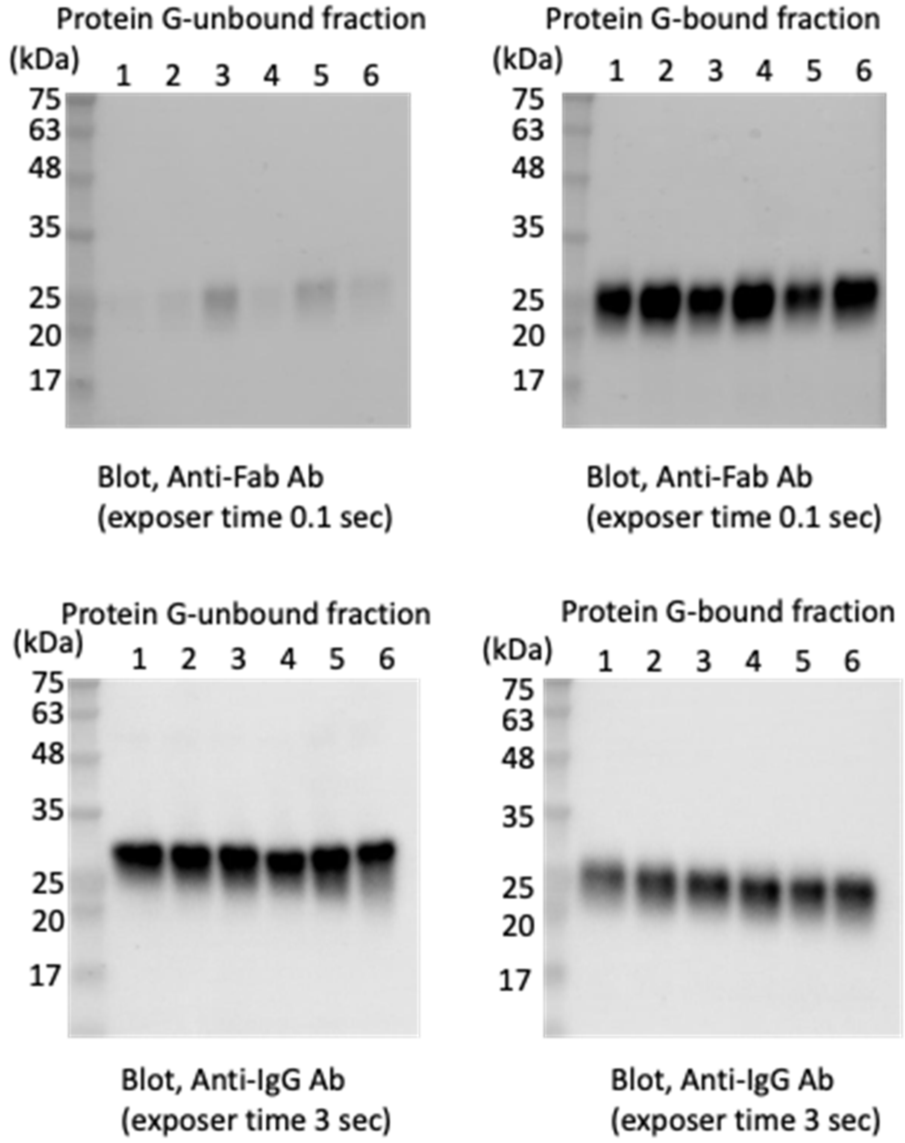

Figure S3. Verification of Fab and Fc fragment separation by immunoblotting. Purified rat IgGs (male: lanes 1–3; female: lanes 4–6) were digested with papain and applied to a protein G column. The unbound and bound fractions were collected and analyzed by immunoblotting using specific to rat Fab and rat IgG. The anti-Fab blot shows signals predominantly in the protein G–bound fraction. In contrast, the anti-IgG blot detected IgG-derived fragments in both fractions, confirming the successful separation of Fab and Fc components.
